# Nudging and boosting children’s restaurant menus for healthier food choice: a blinded quasi-randomized controlled trial in a real life setting

**DOI:** 10.1186/s12889-021-12365-5

**Published:** 2022-01-12

**Authors:** Sven Schneider, Jessica Markovinovic, Jutta Mata

**Affiliations:** 1 Medical Faculty Mannheim, Heidelberg University, Mannheim Institute of Public Health, Social and Preventive Medicine (MIPH), Ludolf-Krehl-Straße 7-11 ZIP, 68167 Mannheim, Germany; 2 School of Social Sciences, Chair of Health Psychology, Mannheim University, L13, 17, 68161 Mannheim, Germany

**Keywords:** Food, Environment, Intervention study, Restaurants, Children

## Abstract

**Background:**

Restaurants are ideal settings for implementing food interventions targeted at children. Studies with adults suggest that changes to the physical menu can lead to healthier food choices; online studies with parents indicate that specific menu designs facilitate healthier choices. However, it is unknown whether applying well-established nudging and boosting methods to children’s menus also increases their choice of healthier meals in a real-world restaurant setting.

**Methods:**

The effects of two versions of a restaurant menu on the frequency of choosing a healthy meal (newly created, healthy target dish) were tested in a blinded quasi-randomized controlled trial. The menu in the control condition contained all dishes (including the healthy target dish) in a standardized format. The intervention menu included nudging (e.g. comic character, fun attractive name for the dish) and boosting elements (e.g. information on low calorie density) next to the healthy target dish. Over five months, the control and intervention menus were switched every two weeks and records were made of how often the healthy target dish was ordered.

**Results:**

In total, 607 orders were made from the children’s restaurant menu (57% from the intervention menu). During the intervention phase, 4.2% of all ordered dishes from the children’s menu were the healthy target dish, during the control phase, 4.4% of orders were for the target dish (p=.896).

**Conclusions:**

Contrary to our hypothesis, a modified children’s menu did not lead to a significant increase in the number of orders for a healthy dish compared with a neutral control menu. Importantly, given that parents and children often choose the child’s dish together, particularly boosting methods that focus on social processes and joint decision making could be promising to increase children’s frequency of healthy food choices in restaurants.

**Trial registration:**

DRKS00027039, registered on 11/22/2021, (Retrospectively registered).

**Supplementary Information:**

The online version contains supplementary material available at 10.1186/s12889-021-12365-5.

## Background

Obesity prevention should start early in life [1]. Educational intervention programs focusing on the individual (e.g. diets, nutrition and exercise programs for children) have long dominated the field, but have not been successful at stopping obesity. Current public health research is increasingly turning to ‘obesogenic environments’ [2], that is, environmental factors that promote obesity through inactivity and unbalanced nutrition. In this context, targeting children’s food environments is an innovative and promising field of prevention [3]. Rideout et al. define the food environment as the sum of all physical, social, economic, cultural, and political factors that impact the accessibility, availability, and adequacy of food within a community or region [3].

Restaurants are important food environments [4–6]. The restaurant sector has reported considerable increases in revenue [7–9]. Families in many countries cook less and commonly consume main meals outside of the home [10]. Therefore, a trip to a restaurant is the perfect setting to expose children to new foods or unfamiliar healthy dishes, with the potential for lasting optical, olfactory, and gustatory experiences. In particular, children’s menus in restaurants could serve as an innovative and potentially significant approach for improving children’s food environments [4, 5].

Two systematic reviews have compiled intervention studies in which an optimized restaurant menu is used to promote healthier food choices. The first review covered 27 studies from 1979 to 2014 [6]. The review’s central findings are that point-of-purchase interventions (interventions using menu labeling) were successful means of promoting healthier food options (that is, newly introduced healthy meals).

However, interventions using typical promotion measures (radio spots, newspaper adverts, posters, leaflets or window signs), training for restaurant owners or service staff, and price reductions were rather unsuccessful. Six of the twenty-seven restaurant interventions included in the review were classified as point-of-purchase interventions. The authors assessed these six interventions with “sufficient evidence” [6]. However, the review authors criticized the fact that only one of these six studies included the use of a control group. It should also be noted that none of the 27 studies included in the review were directed explicitly at children.

The second review exclusively dealt with the type of point-of-purchase intervention in which the restaurant menu was manipulated [11]. This review identified 38 studies from real life settings, conducted from 1976 to 2014. This comparison revealed that easy-to-understand qualitative information such as healthy food symbols, healthier choice tags, or heart symbols were considerably more effective in increasing the choice of healthy options than extensive quantitative information (e.g. calorie, fat and sugar content). However, only 12 of the studies included in this review were conducted in full-service restaurants. Likewise, this review did not include any studies that focused on children’s menus in particular. This review concludes: “Further research could test (…) menu labeling formats (…) by using controlled randomized trials or other designs that include control groups and analyze real-life selection or consumption data before and after menu-labeling interventions” [11]. This is exactly what we have done in the present study. For this purpose, we followed the theoretical and empirical literature that proposes the use of nudging and boosting approaches [12, 13].

*Nudges* are defined as “any aspect of the choice architecture that alters people’s behavior in a predictable way without forbidding any options or significantly changing their economic incentives” [13]. Nudges are shown to have a medium to large effect, especially when used to promote a balanced diet and implemented in a restaurant context [14]. Previous literature suggests some nudging measures for use on children’s menus, such as placing healthy options at the top of the menu where the reader’s gaze typically falls first [15], giving dishes attractive, fun descriptive names [15], and using appropriate children’s comic characters to promote healthy dishes [16]. These types of nudging measures do not require any prior knowledge of nutritional values and are accessible to young visual learners from diverse educational backgrounds [16].

*Boosts* are a different way to influence a person’s decision-making and can be used as an alternative to nudges or jointly with them. Boosts do not target behavior (as nudges do) but rather focus on fostering people’s skills or knowledge or providing decision-making tools or external environments that support people in making their own decisions [12]. An example of a boost on a children’s menu in a restaurant would be an easy to understand label indicating a healthy option on the menu [10, 17] or a contextual statement giving parents additional, generally understandable nutritional information [18, 19].

Restaurants are ideal settings for implementing food interventions aimed at children. Studies of adult cohorts show that interventions that change the physical menu itself are successful. However, to date, only few studies specifically look at children’s menus [10, 15]. The current MINT (Menu INTervention) study aims to investigate whether and how a children’s menu from a real restaurant can be optimized using nudging and boosting techniques to significantly increase orders of a more nutritious target dish (intervention dish) compared with a control menu.

## Methods

### Trial design

**Fig. 1:**
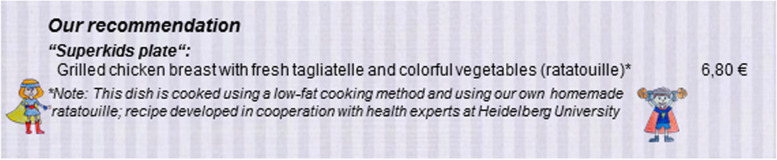
English translation of the description of the target dish on the intervention menu

Between autumn 2018 and spring 2019, we carried out a field experiment in a restaurant in the form of a quasi-randomized trial in which we compared the effect of the restaurant’s regular children’s menu (that now included a new, healthier dish) with a new menu that included both nudging and boosting elements to promote the new healthier dish (see Fig. 1). The project was unconditionally approved by the Medical Ethics Commission II at the Mannheim Medical Faculty of the Heidelberg University (2018-646 N-MA from 20 to 2018). The RCT was pre-registered in the Open Science Framework on 20 November 2018 (https://osf.io/s4t65) and retrospectively registered in the DRKS - German Clinical Trials Register on 22 November 2021 (DRKS00027039).

### Eligibility Criteria for Intervention Restaurant and Investigation Units

The MINT study was carried out in a real life setting in a typical full-service restaurant in the city of Mannheim, Germany. Mannheim lies in western Germany, it has a population of 320,080 inhabitants and covers an area of 145 km² [20, 21]. Mannheim is considered to be a ‘typical major German city’, with a historic city center surrounded by suburbs. This large city was chosen for this study because its social structure is similar to that of the Federal Republic of Germany as a whole.

Based on Ayala’s criteria [4], the restaurant needed to fulfill the following criteria to be included in our study: full-service restaurant; a minimum of 20 tables; offer of local food; and – for coding purposes – sufficiently detailed receipts. The restaurant owner should be 21 years or older, have worked at least 20 h per week for a minimum of 4 months, plan to continue working in the restaurant for the study duration, have decision-making authority, and be willing to provide sales data for research purposes.

The first restaurant that satisfied all of these criteria was selected for the intervention. The restaurant was located close to the city center, featured modern furnishings and had a total indoor seating capacity of 70 with a further 100 seats outside. The restaurant was open from Tuesday-Saturday from 4pm-11pm and on Sunday from 11am-10pm. There were parking spaces for bicycles and cars directly in front of the restaurant as well as a nearby public transport stop with regular services running from early morning until late evening.

Our observations on-site corresponded with the restaurant owner’s description that families were a major part of the clientele and that guests primarily comprised visitors to a popular city park adjacent to the restaurant, residents from the surrounding neighborhoods, as well as adolescent and adult users of the adjacent sports facilities (hockey, gymnastics, climbing, rowing, basketball, endurance sport) owned by one of Mannheim’s biggest sports clubs.

Guests ordered from a typical food menu and were always served at their table by the waiting staff. Prior to the intervention, each of the 36 copies of the menu included a children’s menu that comprised two starters (small soups) and nine main meals for children. It should be noted that the restaurant supported our study at no cost and without any form of return service. As this paper discloses internal operational information and sales figures, we have refrained from explicitly mentioning the name of the cooperating restaurant.

### Study intervention

#### Developing the intervention dish

The first step was to set up a meeting between the restaurant manager, the head chef, the first and the second author and create a new dish, in close coordination with a dietician from the German Federal Research Institute of Nutrition and Food. During this process, care was taken to factor in the relevant quality standards used in Germany when providing catering for children (e.g. in kindergartens and school [22, 23]), including the following criteria: use of lean meat (chicken), low-fat cooking method (grill), use of less heavily processed convenience products (fresh pasta instead of dried), use of fresh vegetables (preparation of raw products) with a cooking method that preserves nutrients (steaming) [22, 23]. One additional criterion was that all elements of the dish should be available over a long period of time at a reasonably cost-effective price. This resulted in a dish that was considered to provide optimum nutrition, namely, grilled chicken breast with fresh tagliatelle and colorful vegetables (ratatouille). This dish was added to the children’s menu for the duration of the experiment.

#### Developing the intervention material

Based on the recommendations taken from previous literature, the following measures were implemented during the experiment: emphasizing the healthiest meal by.

- using a fun and descriptive name (nudging measure based on recommendation from Anzman-Frasca et al. [15] and Basak et al. [16]);
- using comic characters to highlight the meal on the menu (nudging measure based on recommendation from Basak et al. [16] and Lopez et al. [10]);
- listing it first on the menu (nudging measure based on recommendation of Anzman-Frasca et al. [15]) with the information that this dish is recommended by the restaurant (nudging measure based on Cassady et al. [17] and Krukowski et al. [5]).

In addition to this, the menu included a contextual statement that the dish was chosen for its low-fat cooking method, that the ratatouille was not a ready-made sauce but freshly prepared in the restaurant, and that the recipe had been designed in cooperation with a healthcare professional from the University of Heidelberg (boosting measure based on Hobin [18], Lee & Lee [19], Lopez et al. [10] and Krukowski et al. [5]).

#### Cognitive pre-test of intervention material

In the next step, a preliminary evaluation was carried out to assess which name for the dish and which accompanying comic characters (nudging measures) are particularly attractive to children and adolescents. A convenience sample of 13 children and adolescents (age: 10.6 +/- 1.0) were presented with a total of 5 dish names and 6 graphics and asked to select the one that they found to be most attractive.

Most votes went to the name “Superkids Plate” and to a graphic showing one female and one male superhero character. We also asked the participants which descriptions sounded tastiest for the accompanying elements of the dish. The responses to this question led to the decision to describe the side dish as “colorful vegetables (ratatouille)” [*buntes Gemüse (Ratatouille)*].

#### Standard pre-test of intervention material

Next, we carried out a standard pre-test to find out which of the four nudging and boosting measures listed above would be most likely to lead to an increase in orders. To test this, an online survey was set up with a convenience sample of n = 201 participants (age: 32 +/- 8 years) who were asked which dish they would be most likely to order for their children at a restaurant. The survey takers were then shown one of the six following options at random: a children’s menu in which one of the four measures described above was included (Option 1-4), a menu in which all four measures were presented (Option 5) or a menu without any additional measures (Option 6 = control). The intervention dish was selected most frequently when participants were shown Option 5 (31% of all simulated orders), whereas orders were between 24 and 30% when shown the other options. This result led to the decision to use Option 5 for the intervention menu in the field tests (Fig. 1).

### Intervention

The unit of investigation was the number of orders made from the children’s menu. Following the recommendation of Ayala [4], a baseline was established by recording the number of orders from the previous children’s menu, which did not contain the new intervention dish, during a defined baseline period (6-19 November 2018). This was followed by an intervention phase which lasted for five months (20 November 2018-29 April 2019) and was divided into alternating two-week intervention and control phases.

**Fig. 2:**
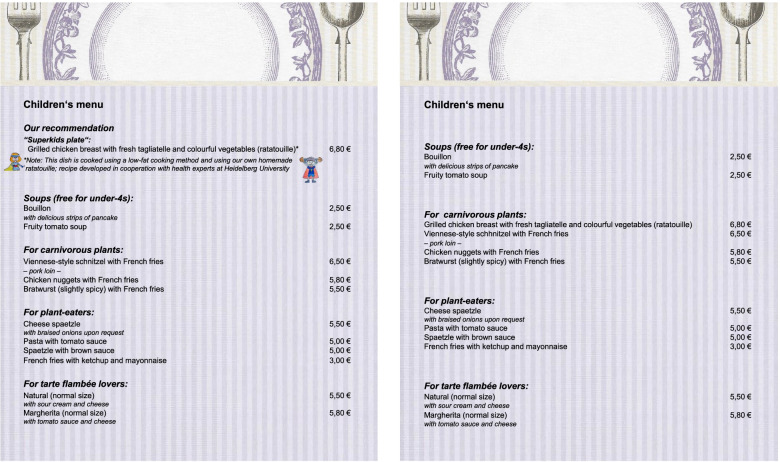
English translation of the intervention menu (left) and the control menu (right)

**Fig. 3:**
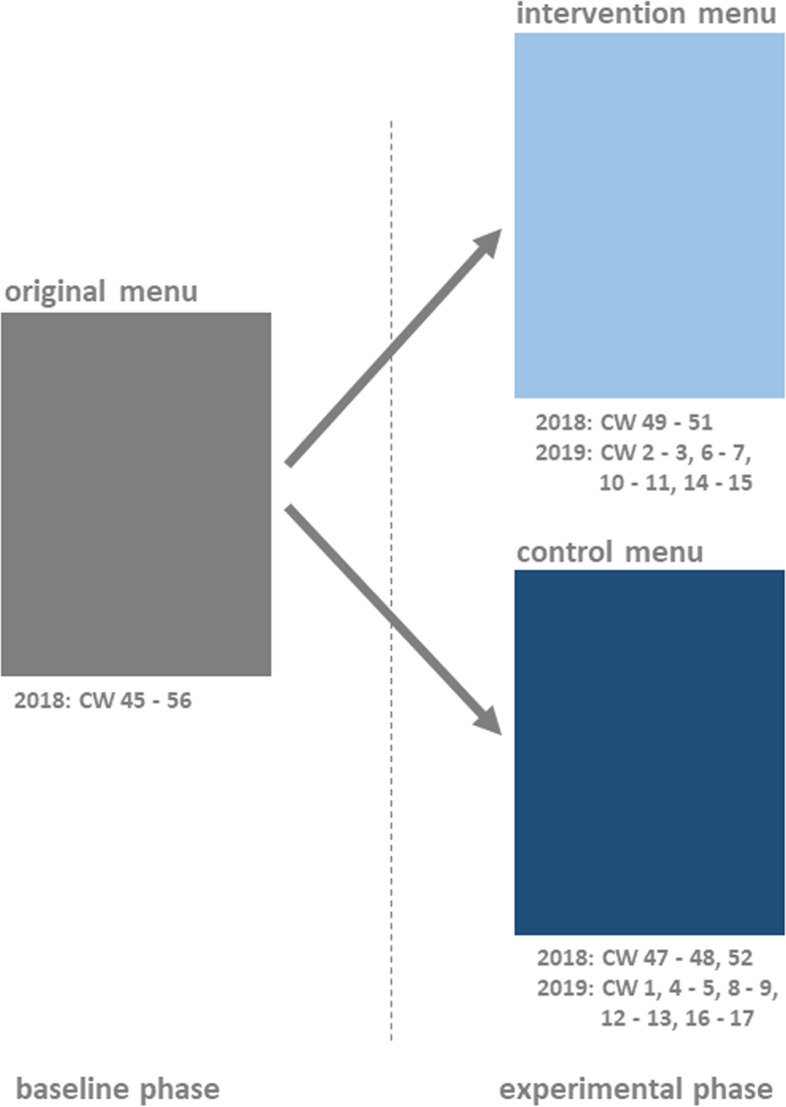
Allocation of the baseline and experimental phases (including intervention and control phases)

During the intervention phases, all of the restaurant’s 36 menus were modified to contain a children’s menu that included the nudging and boosting measures listed above. During the control phases, the second author removed all of the adapted children’s menus from the restaurant menus and replaced them with an unaltered children’s menu in which the new dish was presented in exactly the same way as the other children’s main meals. Figure 2 shows the two different menus and Fig. 3 shows the allocation of the baseline and experimental phases (including intervention and control phases).

### Outcomes

As previously defined and specified in the ethics application and study registry, the primary outcome was the relative number (a/b) of times that the intervention dish was ordered (a) compared with the total number of orders made from the children’s menu (b). This information was obtained from the restaurant owner who, together with the second author, extracted the order data from the restaurant’s POS system at the end of each two-week phase. Every order entered into the POS system includes a unique traceable menu number, date and price, thereby providing objective information regarding the operationalization of our investigation. This corresponds with the standard procedures followed in other studies [4, 6, 10, 11, 15]. Data was likewise extracted from the POS system concerning all order details for the other nine main meals on the children’s menu.

As we were interested in the order behavior concerning typical children’s dishes from a typical children’s menu within the context of a typical restaurant visit (i.e. a hungry child orders a main meal from the children’s menu in a restaurant in interaction with their parents or a responsible adult), we therefore did not include orders for soups or from the main menu in the evaluation. The restaurant had an additional menu for senior citizens and its staff had undergone appropriate training, thereby ensuring that all orders from the children’s menu were made exclusively by children and adolescents. So as not to disrupt the atmosphere in the restaurant and to prevent the setting from being influenced by the presence of one of the field investigators, the children’s menus were replaced and order data was extracted and transferred from the POS system outside of the restaurant’s opening times.

### Quasi-randomization

From a statistical point of view, there was no reason to randomly determine whether the experiment phase began with an intervention phase or a control phase. It seemed reasonable to begin with a control phase to give the restaurant and kitchen staff time to first familiarize themselves with the new dish on the menu and to then familiarize themselves with the newly designed menu in the second phase.

### Blinding

Due to the nature of the study, it was not possible to blind the service staff. As part of the preceding training carried out on-site in the restaurant, the service staff were instructed to give restaurant guests the same treatment in both phases of the study and not to disclose any information about the ongoing investigation. It was thus ensured that the target of the study (the restaurant guest) was blinded to the different possible conditions. In addition to this, the study’s design guaranteed that there was only one version of the menu in circulation at one time and there was no contact between the investigation team and the restaurant guests.

### Data Analysis

#### Confirmatory analysis

All analyses were pre-specified. First, the main research question, whether using the intervention menu (yes/no) lead to more orders of the healthy dish (intervention dish ordered: yes/no), was tested with a two-sided chi-squared test. The underlying hypothesis was specified before the data were collected. The predefined significance level was set at p<.05. All statistical tests were performed with the SPSS for Windows (SPSS Version 25.0, IBM Corp., Armonk, NY, 2017).

#### Additional analyses

For the intervention phase (when intervention and control menu were exchanged bi-weekly), we also conducted an exploratory investigation of the effect of weather conditions on ordering behavior. This was done using data from Germany’s National Meteorological Service. This data was used to carry out non-parametric correlation analyses (Spearman’s Rho ρ) looking at the average temperature during each two week study phase and its correlation with the number of times the intervention dish was ordered during the respective phase.

### Quality Assurance

To ensure the quality of the study and in line with the procedure outlined by Lopez et al. [10], the second author paid several unannounced visits, in addition to the arranged visits for the purpose of exchanging the menus and obtaining order data, to check whether the correct versions were in use. A double entry method was used to set up the data matrix that ensured the quality of the data analysis. In addition to this, all statistical analyses were run twice independently (carried out by the first and the second author). No differences were found.

## Results

The study was carried out as originally planned. There were no problems with exchanging the menus, handling and cooperation between service and kitchen staff. There were no difficulties in extracting and transferring the relevant sales data. There were no unexpected events or other complications.

**Fig. 4:**
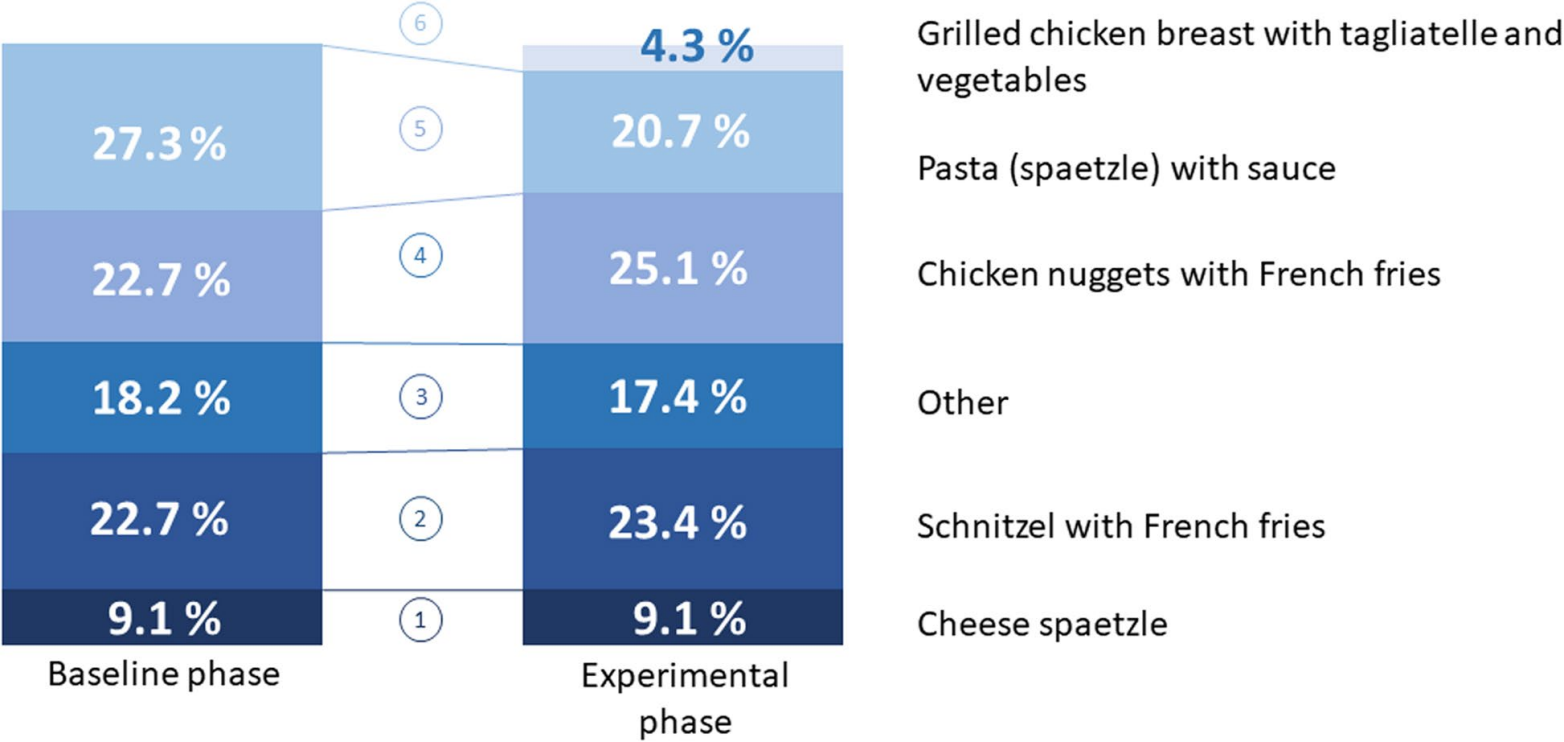
Relative frequency of the number of orders during baseline and experimental phase

Throughout the study period, a total of 607 orders were made from the children’s menu, of which, 585 were made during the experiment phase (of these, n = 335 or 57% were made from the intervention menu and n = 250 from the control menu). During the experiment phase, the intervention dish was ordered 25 times. This represents 4.3% of all orders (see Fig. 4). A comparison between the baseline situation and the experiment phase clearly shows that the introduction of the intervention dish ran parallel to the decline in the number of orders for pasta with sauce, while the relative frequency of the number of orders for the other dishes on the menu remained comparatively stable (see Fig. 4).

**Fig. 5:**
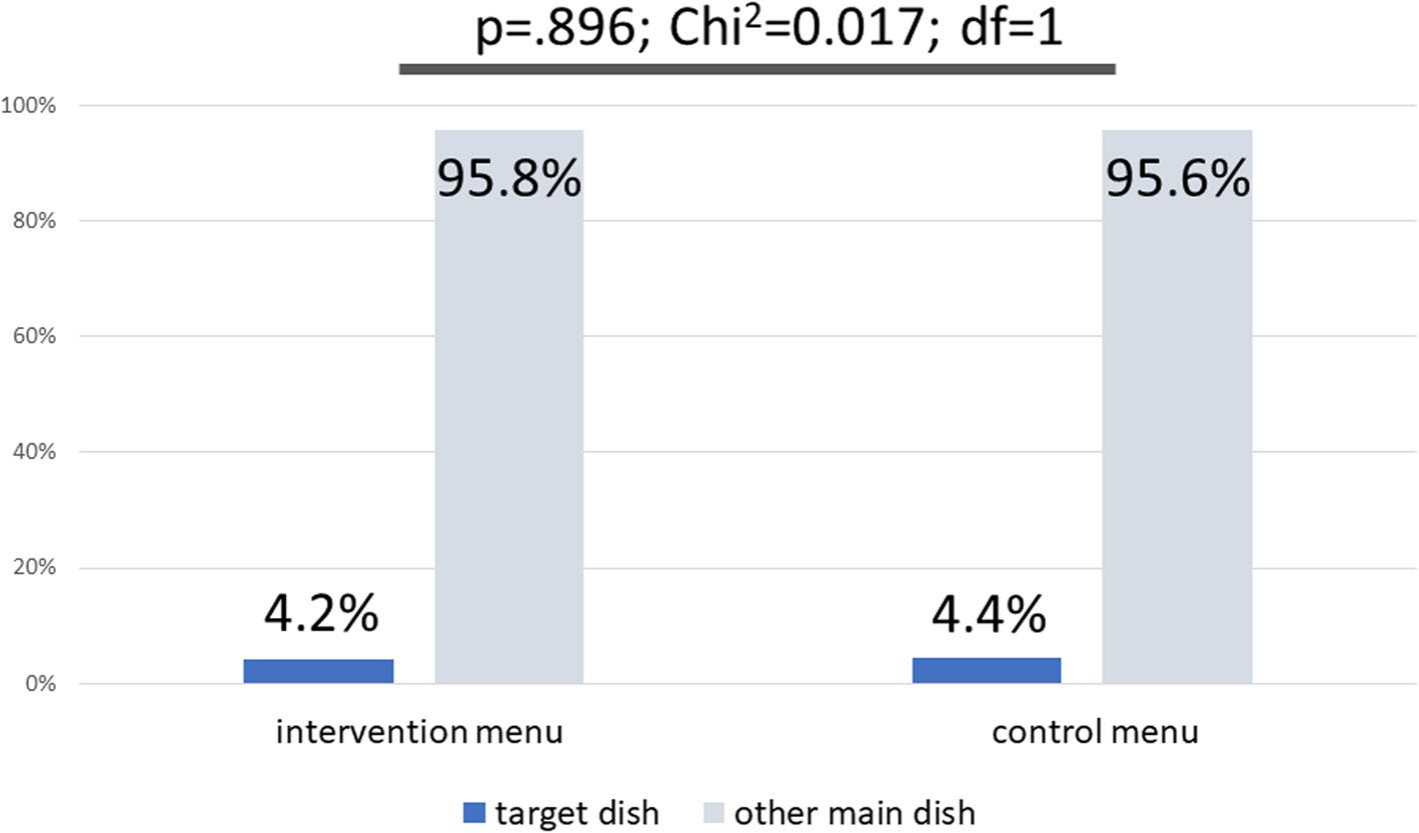
Relative frequency of orders for the target dish using the intervention menu or the control menu

The use of the intervention menu or the control menu did not have a significant effect on the number of orders for the intervention dish (see Fig. 5). During the intervention phases, the number of times the intervention dish was ordered made up 4.2% of all orders (14 of 335 orders) and 4.4% of all orders during the control phase (11 of 250). The difference between these percentage shares was not significant (chi^2^ = 0.017, df = 1, *P* = .90). In addition to this, further analyses showed a clear correlation between the average temperature and both the absolute number and the relative proportion of orders for the intervention dish. During the five-month experiment phase, temperatures ranged from 1.4 to 14 °C (35 to 57 °F). As the temperature increased, not only did the total number of orders increase (Spearman’s rho ρ = 0.731; p=.011) as did the number of times the intervention dish was ordered (ρ = 0.854; p<.001), but more importantly, the relative (!) proportion of times the intervention dish was ordered out of the total orders from the children’s menu also increased (ρ = 0.843; p<.001). In other words – as temperatures rose, the healthy intervention dish (comprising lean meat and steamed vegetables) was ordered more frequently at the expense of other dishes (including a number of deep fried meals).

## Discussion

### Key Findings

Following its introduction, the newly created intervention dish was only ordered once for every twenty orders made. This makes up just 4.3% of all orders made from the children’s menu. This value is considerably lower than was expected on the basis of the pre-test.

However, the most important finding is that the modified menu design, optimized based on recommendations from previous literature, clearly did not affect customer choices. Contrary to our expectations, a children’s menu that had been modified to include boosting and various well-established nudging and elements did not lead to a significant increase in the number of orders for the intervention dish compared with a neutral control menu.

These results are disappointing for us, particularly in light of the additional expense and effort that was required to design and present the new, healthy children’s meal. Besides this, the intervention dish – a more balanced, low-fat and more nutritious dish compared with the other children’s meals – was ordered more frequently on warm, sunny days.

### Comparison with Findings from Other Studies

A synopsis of the current state of research provides a variety of possible explanations for the initially surprising results. The reviews that were discussed in the introduction to this paper only covered studies of adult behavior. To date, only a few intervention studies have been carried out looking at the effects of menu design on children. To the best of our knowledge, the oldest such study was conducted in 2010 and investigated how different menu labeling formats affected parents’ demand for fast food kid’s meals for their children. This study found that when the nutritional information provided was aimed at the parents (i.e. boosting information), this did indeed lead to healthier food choices being made based on the menu options available. However, this study only described a simulated experimental auction with a clinical sample population (n = 99) [24].

Another study from 2016 that likewise employed an experimental auction method and which was only conducted online also led to the same result [18]. In this study, Canadian parents were found to be more likely to order healthier meals for their children when nutritional information (boosting) was included on the simulated children’s menu. Recently, a very similar study emerged from South Korea – here too, parents were provided with different types of nutritional information in the intervention arm of a simulated online experimental auction. This study reported mixed results depending on the type of restaurant and the elements included in the menu [19].

While the three abovementioned studies only reported on simulated situations that did not involve any real ordering, dining and payment processes, the following two studies from the USA did take place in a real-life setting. The intervention arm of the one study employed a similar method to our study, using a combination of nudging and boosting measures (toy incentives, placemats, server prompts, signage) to increase the number of orders of healthy kid’s meals. However, these measures only had a small, not significant effect on the relative sales figures (baseline vs. t_2_: 5% vs. 6%) in full-service restaurants and an unexpected reverse effect in quick-service restaurants (28% vs. 26%, p<.05) [10].

The second study was conducted exclusively in a fast food restaurant. In the intervention arm of this study, a healthy children’s meal was promoted with similar nudging measures to those in our study (including listing the target items prominently at the top of the menu and giving the meal an attractive title, “the Nutty Monkey”). This study also found that participating families did not order the healthy dish more frequently than under control conditions [15].

It clearly seems to make a difference whether the various measures are only employed in a hypothetical situation or in a real-life setting. Both the existing literature and our own pre-test showed that nudging and boosting measures were effective when parents (in the context of a simulation) were able to have full responsibility for deciding what to order for their children, when the order does not have any potential consequences for them.

However, if the order is actually to be served, the nudging and boosting measures employed here clearly fail to make any difference whatsoever. This has previously been observed in two US studies and now also in our study, the first of its kind outside the USA. Importantly though, future research needs to test further nudging and boosting approaches. For example, boosting measures could also aim at building parents’ competences to engage in joint, healthy food-related decision making with their children. Such measures still need to be developed and evaluated.

### Limitations

The primary limitations of this study relate to the minor differences between the pre-test and the intervention material, the limited time covered by the baseline phase, the deliberate decision not to conduct on-site observations, and the extent to which the results can be generalized.

The restaurant had not completed their price calculations at the start of the online pre-tests. We therefore listed the intervention dish with a price of €5.80 throughout the course of the online pre-tests. However, the restaurant management then chose to price the intervention dish at €6.80 on all versions of the menu. This price difference thus limits the extent to which comparisons can be made between the pre-test and the experiment phases. However, it does not have any impact on the results of the main study.

It was necessary to limit the baseline phase to just two weeks owing to internal organization reasons. The comparatively low number of orders made during the baseline phase (n = 22) should be kept in mind when interpreting the data from Fig. 4. This limitation is not significant with regard to the interpretation of the central question of this study (Fig. 5).

A deliberate decision was made not to continuously observe the guests at the restaurant during the field phase, which lasted for several months. German data protection law would not have permitted such observation of underage guests and their parents. Furthermore, it would not have been an economic use of research resources to have a researcher inconspicuously observing customers at every single table in such a large area. It is therefore entirely possible, that children and adolescents ordered alternative meals from the main menu. However, the main research question – which version of the menu lead to the intervention dish being ordered more frequently – was little affected by this limitation. In addition to this, the restaurant staff underwent training which ensured that only children and adolescents ordered from the children’s menu.

Lastly, the findings from this one restaurant in a single city in Germany can by no means be considered to be representative. Nevertheless, it is important to note that the selected restaurant served a typical and demographically varied clientele, providing typical regional cuisine at an average price level.

The main strength of our study is its high degree of external validity. First, unlike other comparable online, clinical, or simulation studies, our intervention took place during the normal operating activities of a real restaurant. As every family was unaware – blinded – that they were automatically included in the study, there was no possibility that participation bias might have an effect. Secondly, our study features a comparatively long study period, spread out over three seasons, as well as a satisfyingly high sample size. Thirdly, both the intervention and control phases were carried out in the same restaurant. Comparable studies have differentiated between intervention and control restaurants leading to confounding influences (differences in location, clientele, premises, staff) or featured several months of pre-post design, during which time seasonal, weather, or economic conditions could change considerably [10]. In contrast, our study circumvented these methodological problems by alternating frequently between intervention and control phases within the same restaurant at short intervals.

## Conclusions

Interventions that involve making changes to children’s menus seem to be a tempting, smart, simple, and cost-effective approach [15]. Our online pre-test led us to expect considerably higher order numbers and indicated that the intervention menu featured a superior design. However, any evidence of the desired effect quickly evaporated when the experiment was subsequently carried out in a real restaurant setting. This seems to indicate that, in addition to how information is presented in a restaurant context, social interactions also play a decisive role in eating-related behavior [25, 26]. When interviewed after visiting a restaurant, parents reported that, in half of all cases, the child made the order decision completely by themselves and that they were at least involved in the decision in a further third of all cases [27]. Our findings indicate that the nature of how parents and children interact with one another at the table when making food-related decisions is an important unknown intermediary factor in this context, i.e. the missing link between stimulus and decision. Future research should focus on these psychological decision-making processes.

## Supplementary Information

**Additional file 1.** Original German version of the intervention menu (left) and the control menu (right).

## Data Availability

The datasets analyzed during the current study are not publicly available as the restaurant owner refrained to disclose operational information and sales figures to the public but are available from the corresponding author on reasonable request.

## Abbreviations

MINT study: Menu INTervention study
POS: Point of sale
