# Supplementary figures and images for: Nudging and boosting children’s restaurant menus for healthier food choice: a blinded quasi-randomized controlled trial in a real life setting
Source: BMC Public Health. 2022 Jan 12;22:78. doi: 10.1186/s12889-021-12365-5 (PMC8756670; doi:10.1186/s12889-021-12365-5)

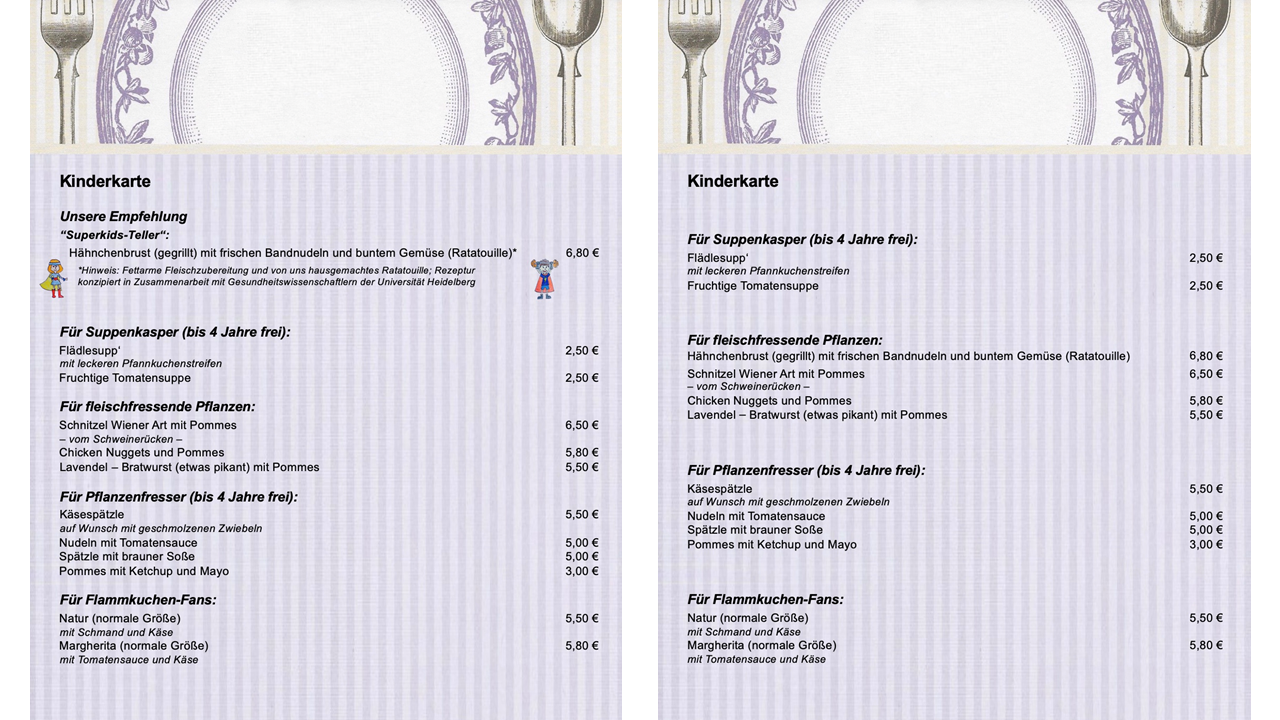

Supplement: Supplementary file 1 — Additional file 1. Original German version of the intervention menu (left) and the control menu (right). [file 12889_2021_12365_MOESM1_ESM.tif]
